# Food Involvement, Eating Restrictions and Dietary Patterns in Polish Adults: Expected Effects of Their Relationships (LifeStyle Study)

**DOI:** 10.3390/nu12041200

**Published:** 2020-04-24

**Authors:** Marzena Jezewska-Zychowicz, Jerzy Gębski, Milena Kobylińska

**Affiliations:** Institute of Human Nutrition Sciences, Warsaw University of Life Sciences (SGGW-WULS), Nowoursynowska 159C, 02-776 Warsaw, Poland; marzena_jezewska_zychowicz@sggw.pl (M.J.-Z.); milena_wasilewska@wp.pl (M.K.)

**Keywords:** dietary patterns, eating restrictions, food involvement, adults, obesity

## Abstract

Understanding the factors that coexist with healthy and unhealthy eating behaviors is prevalent and important for public health. The aim of this study was to investigate the associations between food involvement, eating restrictions, and dietary patterns in a representative sample of Polish adults. The study was conducted among a group of 1007 adults. Questions with the answers yes or no were used to obtain the data regarding eating restrictions. Data relating to food involvement were obtained with the Food Involvement Scale (FIS). Questions from the Beliefs and Eating Habits questionnaire were used to measure the frequency of consumption of different food groups. Five dietary patterns (DPs) were derived using principal component analysis (PCA), i.e., ‘Fruit and vegetables’, ‘Wholemeal food’, ‘Fast foods and sweets’, ‘Fruit and vegetable juices’ and “Meat and meat products’. In each of the DPs, three groups of participants were identified based on tertile distribution with the upper tertile denoting the most frequent consumption. Nearly two-thirds of the study sample declared some restrictions in food consumption. The probability of implementing restrictions in consumption of foods high in sugar, fat and high-fat foods increased in the upper tertile of ‘Fruit and vegetables’ and ‘Wholemeal’ DPs. Moreover, the probability of implementing restrictions in consumption of meat and high-starch products increased in ‘Wholemeal’ DP. The probability of using eating restrictions decreased in the upper tertile of ‘Fast foods and sweets’ and Meat and meat products’ DPs. In conclusion, individuals characterized by high food involvement were more inclined to use eating restrictions than individuals with lower food involvement. Their DPs were also healthier compared to those of individuals manifesting low food involvement. Therefore, promoting personal commitment to learning about and experiencing food may be an effective way of inducing a change of eating habits, and therefore a healthier diet.

## 1. Introduction

Research indicates that a significant proportion of the population, particularly young women, use various dietary restrictions [1,2,3]. This practice may have both positive and negative effects on nutritional status, health, and finally on the quality of life. Eating restrictions may lead to unhealthy dietary habits and eating disorders [4]. On the other hand, present-day diets with a high content of fat, sweet, and energy density often correlate with higher body mass index (BMI) [5,6,7]. Beyond overweight or obesity, such diets can lead to the development of other diet-related chronic diseases, including type 2 diabetes, cardiovascular diseases and some cancers [8]. Therefore, some restraint in consumption of foods, and especially those undesirable in a healthy diet might be beneficial and healthy behavior. For example, calorie restriction provides many benefits for quality of life, especially with respect to loss of weight and fat mass [9,10].

One of the reasons for excessive food consumption and inefficiency in reducing food consumption may be the individuals’ interest in food, i.e., food involvement. According to Bell and Marshall [11] food involvement concerns the extent to which a person cares about and is interested in food. The importance of food in a person’s life varies across individuals, for example, due to socio-demographic characteristics [12]. Differences are also observed in dietary behaviors. Previous studies have shown that consumers who are more food involved are more sensitive to the sensory properties of food [11]. Highly food-involved individuals may be more inclined towards having new food experiences, i.e., food neophilic [13]. Moreover, individuals with higher involvement tend to make healthier food choices [14,15] but they also may reveal both healthier and less healthy dietary practices [16]. It is still unknown whether this relationship stems from being food-involved or whether healthier choices lead to higher food involvement. 

So far, different approaches have been used to measure the construct of food involvement, including but not limited to: a theoretical model of involvement based on expectancy-value theory [17], a large multi-item food-related lifestyle measure [18] or a general involvement measure with the food-provisioning process, the Food Involvement Scale (FIS) [11]. The scale includes preparation, cooking and disposal whichs reflected in the consumers’ engagement with food and their experiences, knowledge, skills and competencies [11]. Thus, the FIS can be considered as a general involvement measure rather than a measure of involvement with a specific food item or brand. For this reason, it appears to be useful in explaining the use of restrictions in food consumption, but also food choices that are revealed in dietary patterns [19,20,21]. The analysis of dietary patterns seems to be a useful approach to explain to what extent healthy choices depend on being more food involved, and also on having greater control over food by using restrictions [22].

Studies have shown that dietary patterns are significantly associated with many disease outcomes or biomarkers, including cardiovascular disease, overweight and obesity, and other diseases [23]. So far, few results have been reported on the association between eating restrictions and dietary patterns [24]. 

We assumed that individuals who are highly involved with food would be less able to use eating restrictions compared to individuals with lower food involvement. For this reason, it seemed more likely that the dietary patterns of more food-involved people will be less healthy. Therefore, the aim of this study was to investigate the associations between eating restrictions, food involvement, and dietary patterns in a representative sample of Polish adults.

## 2. Materials and Methods 

### 2.1. Data Collection

Analyses were carried out on data from the LifeStyle Project. A computer-assisted personal interviewing (CAPI) technique was used in a cross-sectional study. Participants were selected from the panel of the research agency ARC Market and Opinion that consists of approximately 55,000 registered individuals. People are invited to the panel both via off-line and online invitations (65% and 35%, respectively). 

After being sent an invitation to participate in the survey, 6910 people expressed their willingness to participate in the study. Quota selection regarding gender, age, place of residence and region was used to ensure the representativeness of the Polish population. During the recruitment for the study 5 people did not meet the panel criteria, i.e., adults aged 25–65, 144 people stopped filling out the questionnaire before it was completed, 5746 people did not qualify to the quota. During the collection control stage eight people were removed from the database due to very short time of completing the questionnaire and the lack of differences in answers to all frequency questions (Supplementary Materials Figure S1). The study involved 1007 participants.

### 2.2. Ethical Approval

The Ethics Committee of the Faculty of Human Nutrition and Consumer Science, Warsaw University of Life Sciences (SGGW) appointed on the basis of Regulation No. 27 of the SGGW Rector of 5 May 2016 approved the protocol of LifeStyle Study on 27 June 2016, Resolution No. 01/2016 as consistent with the guidelines laid down in the Declaration of Helsinki. Informed consent was provided by participants.

### 2.3. Eating Restrictions

Standardized one-to-one interview (answers yes/no) at respondent’s home was used to obtain data regarding eating restrictions. Questions referred to 10 categories of restrictions, while the analyses included only 6 categories which were indicated by at least 5% of study sample, i.e., restrictions in: food quantity, sugar and/or sweets, high-fat foods, fats, cereals and/or bread and/or potatoes, and meats. Other restrictions concerned: fish, dairy products, raw vegetables and raw fruit.

### 2.4. Dietary Patterns

Questions from Dietary Habits and Nutrition Beliefs Questionnaire–KomPAN [25] were used to measure the frequency of consumption of different food groups, including: wholemeal bread; wholemeal pasta and groats; fermented milk drinks; cheeses (including melted cheese, blue cheese); cured meats and sausages; red meat; white meat; fried foods; fruits; vegetables; vegetable juices; fruit juices; fizzy drinks; meals or snacks such as burgers, pizza, chicken, fries; sweets and cakes; crisps and other salty snacks. To assess the habitual consumption over the past year the question ‘How often do you eat ….? was used for each food group. Food frequency consumption was evaluated in 6 categories: ‘never’ (1), 1–3 times a month (2), once a week (3), few times a week (4), once a day (5), and ‘few times a day’ (6). KomPAN is the questionnaire developed by Polish researchers to evaluate dietary habits, lifestyle and nutrition knowledge whose test-retest reproducibility was assessed among people from all over the country, in healthy subjects and those suffering from chronic diseases [26].

A data-driven (a posteriori) approach was used to identify dietary patterns [23]. PDs were derived by principal component analysis (PCA) to which variables describing frequency of eating some foods were introduced. The factorability of data was confirmed with a Kaiser–Meyer–Olkin (KMO) measure of sampling adequacy and Bartlett’s test of sphericity which achieved statistical significance [27]. KMO value was 0.781 which indicates the correct choice of analysis and the number of factors. Bartlett’s test had a significance of *p* < 0.0001. To derive dietary patterns a varimax normalized rotation was used in order to extract not correlated factors and obtain high variance explained [27]. Eigenvalues of at least 1.00 were considered. Five dietary patterns were derived: ‘Fruit and vegetables’, ‘Wholemeal food’, ‘Fast foods and sweets’, ‘Fruit and vegetable juices’ and ‘Meat and meat products’. Total variance explained was 64.2%. The relationship between the so-identified dietary patterns and selected elements of the lifestyle was already presented in other articles [28,29].

### 2.5. Food Involvement 

Data relative to food involvement were obtained with the Food Involvement Scale developed in 2002 by Bell and Marshal [11]. The original scale includes 12 items with a Likert scale ranging from (1) “completely disagree” to (7) “completely agree”. In this study, the interval between the possible answers was reduced from 7 to 5 points (1—disagree; 2—rather disagree; 3—neither agree nor disagree; 4—rather agree; 5—agree). The original version was translated to Polish and then the instrument was back-translated from Polish to English. Negative statements from original scale were changed to positive statements (without “no”). The Food Involvement score was calculated by the sum of the individual’s answers. The higher the score, the greater was the food involvement of the person. The scores obtained ranged from 13 to 60 points.

### 2.6. Sociodemographic Variables

Considered sociodemographic variables were as follows: gender, age, education, place of residence. Body mass index (BMI) was calculated using self-reported weight and height and categorized according to International Obesity Task Force (IOTF) standards [30].

### 2.7. Statistical Analysis

Participants in each of the dietary patterns were divided into three groups based on tertile distribution: 1st tertile (T1), 2nd tertile (T2) and 3rd tertile (T3). T3 had the strongest adherence to the pattern while T1 had the weakest one. Median was used to categorize the sample in accordance to food involvement. Scores on the Food Involvement Scale higher than 40 points indicate high food involvement. 

Associations between eating restrictions, food involvement and dietary patterns were verified using logistic regression analysis. Odds ratios (ORs) represented the probability of the adherence to tertiles of each dietary pattern. The reference groups (OR = 1.00) were those who declared that they did not follow the restrictions. Wald’s test was used to assess the significance of ORs. Tests of linear trend across increasing tertiles of dietary pattern adherence (for ORs) were calculated for each type of eating restriction. *P*-value < 0.05 was considered as significant for all tests. All analyses were carried out applying sample weights to adjust for non-response and missing data. All analyses were performed using SAS 9.4. software (SAS Institute, Cary, NC, USA).

## 3. Results

### 3.1. Sample Characteristics

The sample consisted of 1007 participants (529 women and 478 men) aged 21 to 65 years. Among respondents, 35.7% were overweight and 12.7% were obese, that is their BMI calculated on the basis of declared height and weight was 25 and above. Table 1 displays characteristics of the study sample. 

### 3.2. Dietary Patterns

The characteristics of the identified dietary patterns, i.e., ‘Fruit and vegetables’, ‘Wholemeal food’, ‘Fast foods and sweets’, ‘Fruit and vegetable juices’ and ‘Meat and meat products’, are summarized in Table 2 [28,29].

### 3.3. Eating Restrictions

Nearly two-third (66.4%) of the study population declared following some restrictions in food consumption, of this 11.7% declared continuous use of the restrictions, and 54.7% occasional use.

**Figure 1:**
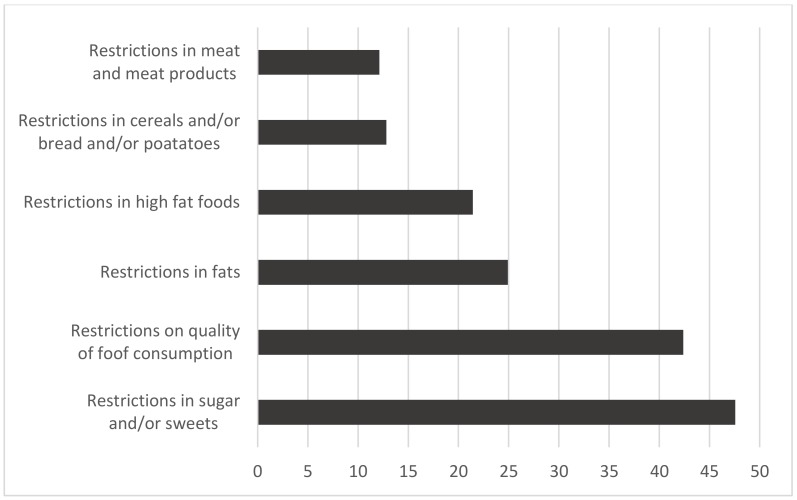
Eating restrictions in the sample (%).

The types of eating restrictions are described in Figure 1. Above two-fifths of the sample (42.4%) declared following restrictions regarding the quantity of consumed foods. In the total sample, the most common restrictions regarded consumption of sugar and/or sweets (47.6%), fats (24.9%), and high-fat foods (21.4%).

### 3.4. Food Involvement

The mean value of the food involvement was 40.29, standard deviation—7.64 and median—40. The items included in the FIS are described in Table 3.

### 3.5. Associations between Eating Restrictions, Food Involvement, and Dietary Patterns

The associations between variables are described in Table 4 and Table 5. People in the upper tertile of ‘Fruit and vegetables’ compared to those in the bottom tertile of this DP were more likely to follow restrictions regarding quantity of consumed food (OR: 2.30, 95% confidence interval (CI) 1.66–3.19) and restrictions in consumption of sugar and/or sweets (OR: 1.89, 95% CI 1.36–2.61), fats (OR: 1.93, 95% CI 1.34–2.77), and high-fat foods (OR: 1.90, 95% CI 1.29–2.80). People in the upper tertile of ‘Wholemeal food’ compared to those in the bottom tertile of this DP were more likely to follow all included restrictions, i.e., restrictions regarding quantity of consumed food (OR: 1.76, 95% CI 1.27–2.45), restrictions in consumption of sugar and/or sweets (OR: 3.14, 95% CI 2.25–4.39), meat (OR: 3.13, 95% CI 1.83–5.35), fats (OR: 2.05, 95% CI 1.40–2.99), high-fat foods (OR: 1.78, 95% CI 1.20–2.65), and cereals and/or bread and/or potatoes (OR: 1.91, 95% CI 1.20–3.04).

People in the upper tertile of ‘Meat and meat products’ compared to those in the bottom tertile of this DP were less likely to follow restrictions in consumption of sugar and/or sweets (OR: 0.64, 95% CI 0.49–0.88), fats (OR: 0.68, 95% CI 0.47–0.98), high-fat foods (OR: 0.58, 95% CI 0.39–0.88) and meat (OR: 0.29, 95% CI 0.17–0.48). Whereas people in the upper tertile of ‘Fast food and sweets’ compared to those in the bottom tertile of this DP were less likely to follow all restrictions with exception of restriction in consumption of meat. Participants in the upper tertile of ‘Fruit and vegetables juices’ compared to those in the bottom tertile of this DP were less likely to follow restrictions regarding quantity of consumed food (OR: 0.61, 95% CI 0.44–0.85). 

People in the upper tertiles of all DPs, with exception of ‘Fast food and sweets’, were more likely to be highly food involved compared to those in the bottom tertiles of these DPs. In the upper tertile of ‘Wholemeal food’ were more than threefold more likely to be highly food involved compared to the bottom tertile of this DP (more than 40 points in FIS) (Table 3).

Respondents who were highly food involved (FIS > 40 points) were more likely to declare restrictions in consumption of fats (OR: 1.35, 95% CI 1.01–1.80) and high-fat foods (OR: 1.64, 95% CI 1.21−2.23) (Table 4).

## 4. Discussion

The results of our study have shown that implementation of eating restrictions is a common practice, with over 60% of the participants reporting this behavior. Approximately 12% of respondents declared continuous use of restrictions in their diet. Thus, the results obtained in the group of Polish girls and young women [24] have been confirmed in the adults. It is a challenge for many individuals to practice dietary restriction, especially caloric restriction in an obesogenic environment so conducive to overeating [31]. A high percentage of people who declared restrictions points to the importance of this issue. Understanding the causes of these behaviors and their consequences for the diet should be deepened in further studies, especially since such practices are controversial. Eating restrictions may lead to unhealthy dietary habits and eating disorders [32]. On the other hand, moderate restraint in consumption of foods undesirable in a healthy diet and practising caloric restriction in the obesogenic environment could be considered as a beneficial and health promoting behavior [31]. 

Our findings have shown that restrictions in food quantity as well as those related to foods undesirable in a healthy diet (i.e., sugar, sweets, fats) were associated with healthy dietary patterns, mainly ‘Fruit and vegetables’ and ‘Wholemeal food’ DP. Participants who have reported restricting the overall quantity of food as well as sugar and/or sweets, fat, and foods high in fat adhered to the upper tertile of these DPs. Avoidance of foods high in fat and sugar by consumers who declared high intakes of fruit and vegetables has been previously reported [24,33]. Thus, this may be considered as evidence confirming the positive effects of food restrictions. Participants who restricted sugar and/or sweets and meat were more likely (approximately three times) to adhere to ‘Wholemeal foods’ DP, than those who restricted quantity of food, fats, high-fat foods, and starchy foods such as cereals, bread and potatoes. Because the awareness of the importance of a healthy lifestyle is increasing, breads containing whole grain, multi-grain, or functional components, such as fiber, attract attention of some consumers [34,35]. The higher health awareness of these people can explain their practices related to limiting sugar and/or sweets [36] and meat [37]. Restrictions regarding starchy foods in people from the upper tertile of ‘Wholemeal foods’ DP may result from using substitution of products originating from the refined flour (white bread, some cereals). Greater availability of wholemeal cereal products and the fact that these products meet more sensory expectations of consumers greatly facilitate such replacement [38].

Unhealthy dietary behaviors were found in participants from the upper tertile of ‘Fast foods and sweets’ DP, similarly to the research of Wadolowska et al. [39] carried out in young females. This may indicate the prevalence of this type of behavior in the Polish population, regardless of the age and gender. As expected, respondents with the highest adherence to this DP were less likely to restrict high-fat foods, sugar and sweets [24], but also fats and quantity of food. A lower tendency to limit consumption of such foods may indicate little or no interest in the quality of the diet and strong preferences for highly palatable foods. Similar results were obtained by French et al. [40] who found that higher frequency of fast foods consumption was more prevalent in women with low dietary restraint. Apart from low likelihood of restrictions in food quantity and foods rich in sugar and fat, in ‘Fast foods and sweets’ pattern we also observed a lower likelihood to restrict starchy foods such as cereals, bread and potatoes. In that context, absence of restrictions in unhealthy foods should be considered as a potential reason for excessive energy intake, especially because of the amount of energy, fat and sugar supplied with the diet. Many studies have linked low restraint with overweight and obesity [41,42]. 

Participants from the upper tertile of ‘Meat and meat product’ DP were less likely to restrict the consumption of majority of food products, mainly meat (by 71%), as well as fat (by 32%) and high-fat foods (by 42%). This finding confirms the similarity of food restrictions found for the “traditional Polish” pattern in the study of Galinski et al. [24]. Meat and meat dishes have always been and still are an important component of the traditional diet of Poles.

Dietary restrictions did not differentiate the probability of adherence to the upper tertile of the ‘Fruit and vegetable juices’ DP with one exception. Only in the case of restriction in food quantity, the adherence to the upper tertile of this DP was significantly lower compared to those from the bottom tertile (by 39%). Lack of restrictions may indicate a lack of behavioral control in a group of people who often drink juices. It may cause health risk because fruit juice is a source of sugar and low in fiber, which predisposes to weight gain and obesity [43], moreover drinking juice may replace eating fresh vegetables and fruits. 

Frequent consumption of food characteristic for all DPs was more likely in people who were more food involved with one exception, the ‘Fast food and sweets’ DP. A higher intake of fruit and vegetables among individuals with a higher FIS was also reported by Marshall and Bell [14]. Nevertheless, by contrast with our research, they have found that highly food involved individuals were less likely to consume snack products. In our study people with higher food involvement were beyond threefold more likely to often consume wholemeal food compared to those in the bottom tertile of this DP which strongly confirms the relationship between food involvement and healthy diet. Nevertheless, our results only partially confirm that higher food involvement promotes more healthy diet [14] due to frequent consumption of meat and its products. This is also confirmed by the lack of a negative association with the consumption of fast food. Our results are, therefore, more consistent with those from the studies by Sarmugan and Worsley [16] who pointed out that consumers with higher levels of food involvement may be characterized by both healthier and less healthy dietary practices.

Nonetheless, participants who were more food involved (FIS > 40 points) were more likely to apply restrictions in fats and high-fat foods, which may be conducive to reducing energy intake. It is also confirmed by Marshall and Bell [14] who indicated that military personnel with higher FIS exhibited lower caloric intake, of which a lower proportion was derived from fat. 

Most previous studies suggested that higher levels of food involvement appear to be associated with healthier dietary behavior [15,44] and we confirmed it partially. But it is also known that lower food involvement is associated with higher convenience orientation [45]. Because both the results of previous studies and of our research indicate some inconsistencies in relationship between food involvement and healthy food choices further research should be encouraged. Future studies should include also other factors conditioning food choice and eating behaviors, for example convenience which was not taken into account in our research.

The limitations of our study relate to the potential biases that may occur when self-reported data is analyzed. The most common biases in self-reported data are related to selective memory, telescoping, attribution, and exaggeration [46]. However, the main strength is a relatively large representative sample of the Polish population. The use of the questionnaire is also limited due to overestimation of some foods’ consumption when Food Frequency Questionnaires (FFQ) is used. We have chosen FFQ because we aimed to see predominantly ‘healthy’ and ‘unhealthy’ dietary patterns, rather than exact amount of foods. Although our findings should not be generalized to the population with different cultural background, the study provides an interesting insight into dietary restrictions and their association with dietary patterns. The results can be used in the preparation of interventions targeted at health-related changes in existing dietary patterns.

## 5. Conclusions

Eating restrictions are a common practice among Poles, as they were used by over two thirds of the study sample. Declared restrictions in the consumption of foods high in sugar, fat and high-fat foods were observed in a group of people who often ate fruit, vegetable and wholemeal products. In addition, people who often consumed whole grains applied restrictions regarding eating meat and high-starch products. Such restrictions can be considered beneficial for health and interpreted as avoidance of foods that are not desirable in a healthy diet, especially when consumed in excess. There were no such restrictions in unhealthy dietary patterns, i.e., ‘Fast foods and sweets’ and Meat and meat products’. It could mean that the self-regulating behaviors do not occur among these adults. Further research should be focused on seeking explanations on why people who often eat sweets, fast food and meat are less likely to restrict less healthy food in their diet. It can be stated that dietary restrictions of sugar, high-fat foods and fats can be considered as predictors of healthy dietary patterns in the population of Polish adults. 

Food involvement can be considered a factor that positively correlates with the use of food restrictions. However, participants with higher food involvement were more likely to use restrictions only regarding fats and high-fat foods, which may be conducive to reducing energy intake. Thus, higher level of food involvement appears to be associated with healthier dietary behavior. However, less healthy dietary practices may also be observed among more food-involved people, i.e., frequent consumption of meat. Some existing inconsistencies in relationship between food involvement and healthy food choices raise new questions, therefore further research should be encouraged. Future studies should include also other factors conditioning food choice and eating behaviors, for example convenience which was not taken into account in our research.

In conclusion, it should be stated that our assumption that individuals who are more food involved would be less able to use eating restrictions in comparison to less food-involved individuals was incorrect. Moreover, our results did not confirm that the dietary patterns of more food involved people are less healthy. We assumed that the intensity of the contact with food (high food involvement) encourages its consumption, and therefore makes it difficult to limit. The lack of confirmation of this assumption may suggest that food involvement associates rather with greater nutritional awareness, resulting in healthier dietary patterns in people who are more food involved. This may be relevant for creating public health policies: the combination of nutritional education with a greater personal commitment to learning about and experiencing food [47,48,49] could be more effective in inducing the change of diet to a healthier one.

## Figures and Tables

**Table 1:** Sample characteristics.

Variables		N = 1007	%
Gender	Female	529	52.5
	Male	478	47.5
Age	21–34 years	370	36.7
	35–44 years	235	23.3
	45–54 years	132	13.1
	55–65 years	270	26.8
Residence	City	539	53.5
	Town	199	19.8
	Country side	269	26.7
Education	Secondary and lower than secondary	403	40.1
	Higher	604	59.9
Body mass index (BMI) category	Underweight (BMI ≤ 18.5 kg/m^2^)	35	3.5
	Normal weight (18.5 kg/m^2^ < BMI ≤ 25 kg/m^2^)	484	48.1
	Overweight (25 kg/m^2^ < BMI ≤ 30 kg/m^2^)	360	35.7
	Obesity (BMI > 30 kg/m^2^)	128	12.7

N—number of participants

**Table 2:** Factor-loading matrix for the dietary patterns identified by principal component analysis (PCA).

Variables	Factor 1 Fast Foods and Sweets	Factor 2 Meat and Meat Products	Factor 3 Fruit and Vegetable	Factor 4 Whole Meal Food	Factor 5 Fruit and Vegetable Juices
Crisps and other salty snacks	0.824	.	.	.	.
Meals or snacks such as burgers, pizza, chicken, fries	0.756	.	.	.	.
Sweets and cakes	0.702	.	.	.	.
Fizzy drinks	0.633	.	.	.	.
Red meat (pork, beef, venison)	.	0.783	.	.	.
White meat (poultry, turkey)	.	0.748	.	.	.
Cured meats and sausages	.	0.696	.	.	.
Fried foods	.	0.551	.	.	.
Fruits	.	.	0.825	.	.
Vegetables	.	.	0.764	.	.
Cheeses (including melted cheese, blue cheese)	.	.	.	.	.
Whole meal pasta, groats	.	.	.	0.839	.
Whole meal bread	.	.	.	0.763	.
Fermented milk drinks	.	.	.	.	.
Vegetable juice	.	.	.	.	0.830
Fruit juice	.	.	.	.	0.799
Variance Explained (%)	24.9	16.0	9.5	7.4	6.4
Total Variance Explained (%)	64.2				
Kaiser’s Measure of Sampling Adequacy:	0.781				

Factor loadings of ≤|0.50| are not shown in the table for simplicity.

**Table 3:** Food Involvement Scale (FIS) characteristics.

Food Involvement Scale (FIS)	Mean	SD	Median
I think a lot about food each day.	3.01	1.02	3
Cooking or barbequing is a lot of fun.	3.55	1.01	4
Talking about what I ate or am going to eat is something I like to do.	3.27	1.03	3
Compared with other daily decisions, my food choices are very important.	2.67	1.04	3
When I travel, one of the things I anticipate most is eating the food there.	3.45	0.99	4
I do most or all of the clean up after eating.	3.66	0.93	4
I enjoy cooking for others and myself.	3.53	1.07	4
When I eat out, I think or talk a lot about how the food tastes.	3.27	0.98	3
I do like to mix or chop food.	3.28	1.07	3
I do most or all of my own food shopping.	3.44	1.16	4
I wash dishes or clean the table.	3.39	1.15	4
I care whether or not the table is nicely set.	7.74	0.90	4

SD—standard deviation.

**Table 4:** Odds ratio (OR (95% confidence interval (CI)) of dietary patterns by eating restrictions in the sample.

Variable	Fast Foods and Sweets			Meat and Meat Products			Fruit and Vegetables			Whole Meal Food			Fruit and Vegetables Juices		
	T1	T3	*p^a^*	T1	T3	*p*	T1	T3	*p*	T1	T3	*p*	T1	T3	*p*
Restriction on quantity of food consumption (ref.: without restrictions):															
OR crude (95%CI)	1	0.41 (0.30; 0.56)	****	1	0.94 (0.68; 1.30)	ns	1	2.30 (1.66; 3.19)	****	1	1.76 (1.27; 2.45)	***	1	0.61 (0.44; 0.85)	**
Restrictions in consumption of sugar and/or sweets (ref.: without restrictions):															
OR (95%CI)	1	0.35 (0.25; 0.48)	****	1	0.64 (0.49; 0.88)	***	1	1.89 (1.36; 2.61)	****	1	3.14 (2.25; 4.39)	****	1	0.88 (0.63; 1.22)	ns
Restrictions in consumption of fats (ref.: without restrictions):															
OR (95%CI)	1	0.42 (0.29; 0.61)	****	1	0.68 (0.47; 0.98)	*	1	1.93 (1.34; 2.77)	***	1	2.05 (1.40; 2.99)	***	1	0.89 (0.61; 1.28)	ns
Restrictions in consumption of high-fat foods (ref.: without restrictions):															
OR (95%CI)	1	0.23 (0.15; 0.36)	****	1	0.58 (0.39; 0.88)	***	1	1.90 (1.29; 2.80)	***	1	1.78 (1.20; 2.65)	**	1	1.13 (0.77; 1.67)	ns
Restrictions in consumption of cereals and/or bread and/or potatoes (ref.: without restrictions):															
OR (95%CI)	1	0.47 (0.30; 0.74)	****	1	1.16 (0.74; 1.83)	ns	1	0.80 (0.51; 1.27)	ns	1	1.91 (1.20; 3.04)	**	1	0.91 (0.57; 1.43)	ns
Restrictions in consumption of meat (ref.: without restrictions):															
OR (95%CI)	1	0.71 (0.44; 1.14)	ns	1	0.29 (0.17; 0.48)	****	1	1.32 (0.81; 2.15)	ns	1	3.13 (1.83; 5.35)	****	1	1.54 (0.94; 2.52)	ns
Food involvement (ref. higher than 40 points)															
OR (95%CI)	1	1.30 (0.94; 1.79)	ns	1	1.80 (1.30; 2.49)	***	1	1.85 (1.34; 2.57)	***	1	3.46 (2.48; 4.82)	****	1	2.00 (1.44; 2.77)	****

^a^ statistically significant: * *p* < 0.05, ** *p* < 0.01, *** *p* < 0.001, **** *p* < 0.0001, ns—statistically insignificant (Wald’s test); T1—the bottom tertile; T3—the upper tertile (T3—the most frequent consumption, while T1—the lowest consumption).

**Table 5:** Odds ratio (OR (95% CI) of food involvement by eating restrictions in Polish sample.

Eating Restrictions:	Food Involvement		
	FIS ≤ 40 Points	FIS > 40 Points	*P^a^*
Restriction on quantity of food consumption (ref.: without restrictions):			
OR crude (95%CI)	1	1.06 (0.86; 1.42)	ns
Restrictions in consumption of sugar and/or sweets (ref.: without restrictions):			
OR (95%CI)	1	1.28 (0.96; 1.57)	ns
Restrictions in consumption of fats (ref.: without restrictions):			
OR (95%CI)	1	1.35 (1.01; 1.80)	*
Restrictions in consumption of high-fat foods (ref.: without restrictions):			
OR (95%CI)	1	1.64 (1.21; 2.23)	**
Restrictions in consumption of cereals and/or bread and/or potatoes (ref.: without restrictions):			
OR (95%CI)	1	1.32 (0.91; 1.92)	ns
Restrictions in consumption of meat (ref.: without restrictions):			
OR (95%CI)	1	1.27 (0.87; 1.86)	ns

^a^ statistically significant: * *p* < 0.05, ** *p* < 0.01 ns—statistically insignificant (Wald’s test); FIS—Food Involvement Scale score.

## Supplementary Materials

The following are available online at https://www.mdpi.com/2072-6643/12/4/1200/s1, Figure S1: Flow-chart of participants.
