# Supplementary figures and images for: Food Involvement, Eating Restrictions and Dietary Patterns in Polish Adults: Expected Effects of Their Relationships (LifeStyle Study)
Source: Nutrients. 2020 Apr 24;12(4):1200. doi: 10.3390/nu12041200 (PMC7230548; doi:10.3390/nu12041200)

Figure S1: Flow-chart of participants

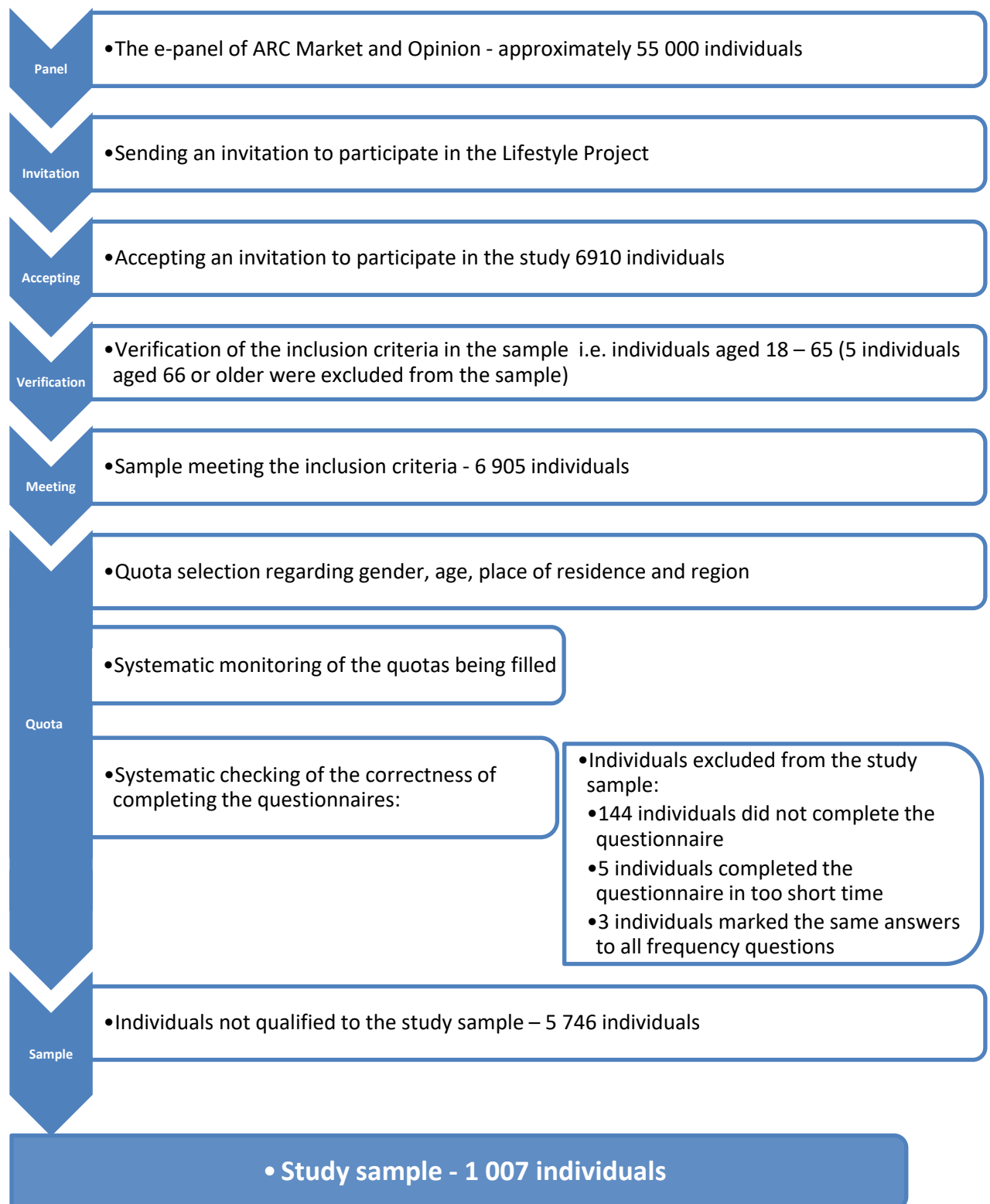

Supplement: Supplementary file 1 [file nutrients-12-01200-s001.pdf]
